# Identification of Leaf Stripe Resistance Genes in Hulless Barley Landrace Teliteqingke from Qinghai-Tibet Plateau

**DOI:** 10.3390/ijms26031133

**Published:** 2025-01-28

**Authors:** Zemin Tan, Sai Zhang, Yunfeng Qu, Shenghua Kang, Shiyu Fang, Lu Hou

**Affiliations:** 1 Qinghai Academy of Agriculture and Forestry Sciences, Qinghai University, Xining 810016, China; 18607329514@163.com (Z.T.); x1652251611@163.com (S.Z.); 18685188795@163.com (S.K.); fsy19960524@163.com (S.F.); 2 Laboratory for Research and Utilization of Qinghai Tibet Plateau Germplasm Resources, Xining 810016, China; 3 Key Laboratory of Agricultural Integrated Pest Management, Xining 810016, China; 4 Institute of Biotechnology, Xianghu Laboratory, Hangzhou 311231, China; quyunfeng@xhlab.ac.cn

**Keywords:** hulless barley, leaf stripe disease, RNA-seq, SNP, gene mapping

## Abstract

Leaf stripe disease, caused by *Pyrenophora graminea*, is a seed-borne fungal disease that significantly impacts hulless barley (*Hordeum vulgare* var. *nudum*) production on the Qinghai-Tibet Plateau. This study aimed to identify genetic factors conferring resistance to the leaf stripe by analyzing an F_3_ population derived from a cross between the resistant landrace Teliteqingke and the susceptible landrace Dulihuang. Genetic analysis revealed that resistance in Teliteqingke was governed by two dominant genes. Using bulked segregant analysis combined with an SNP array (BSA-SNP) and RNA-seq, we identified two candidate regions on chromosomes 3H and 7H. Further analysis focused on chromosome 3H, which revealed a candidate genomic region containing seven potential disease-resistance genes. Among these, RT-qPCR experiments demonstrated significant expression induction of *HORVU.MOREX.r3.3HG0232110.1* (encoding a RING/U-box superfamily protein) and *HORVU.MOREX.r3.3HG0232410.1* (encoding a bZIP transcription factor) showed significant expression induction following inoculation with *P. graminea*. These genes are strong candidates for the resistance mechanism against leaf stripes in Teliteqingke. These results provide a foundation for functional validation of these genes and offer valuable insights for breeding disease-resistant hulless barley.

## 1. Introduction

Hulless barley (*Hordeum vulgare* L. var. *nudum*), also known as naked barley, is a dietary staple on the Qinghai-Tibet Plateau, where it constitutes 95% of cultivated barley [1]. Known for its short growing cycle and adaptability to harsh climates, hulless barley is crucial for food security in the region. However, it is highly susceptible to barley leaf stripe disease, a seed-borne fungal disease caused by *Pyrenophora graminea* Ito and Kuribayashi [anamorph: *Drechslera graminea* (Rabenh.) Shoemaker]. Severe outbreaks can reduce yields by 10–25% [2]. Since its first documented in eastern Tibet in the late 1970s [3], the disease has spread extensively, posing significant threats to food security and the economic stability of farming communities.

Barley leaf stripe spreads through fungal mycelia that colonize seeds, residing between the pericarp, hull, and seed coat. Upon seed germination, the fungus initiates a new infection cycle [4,5]. Chemical seed treatments are commonly used to manage the disease, but they can negatively impact the fragile ecosystems of the Qinghai-Tibet Plateau. Developing disease-resistant hulless barley varieties offers an environmentally sustainable alternative to chemical pesticides. This approach reduces environmental harm by preserving soil health and water quality, lowers production costs, and improves yield stability. It benefits both the environment and local farming communities [6]. Sustainable disease management through resistant varieties ensures long-term agricultural productivity and economic resilience.

Understanding the genetic basis of disease resistance is crucial for developing barley varieties resistant to leaf stripe disease. Genetic mapping has identified specific genes and quantitative trait loci (QTL) associated with this trait. The first such gene, *Rdg1a*, was mapped to the long arm of chromosome 2H in the barley cultivar Vada, which also carries the powdery mildew resistance gene *MlLa*, derived from *H. laevigatum* L. [7]. *Rdg1a* was later found in *H. spontaneum* C. Koch., a wild barley relative [8]. Another resistance gene, *Rdg2a*, was located on the short arm of chromosome 7H and was precisely mapped using high-resolution techniques [9]. Additionally, Si et al. [10] characterized *Rdg3*, a distinct leaf stripe resistance gene located on chromosome 7HS. These discoveries demonstrate the genetic diversity that contributes to barley’s resistance to leaf stripe disease.

QTL research has further elucidated the genetic architecture of barley’s resistance. Studies using bi-parental populations, derived from crosses of two parental lines, have been crucial in detecting resistance QTL. For example, Pecchioni et al. [11] identified a QTL on chromosome 7H conferring partial resistance in the Proctor × Nudinka population. Similarly, Arru et al. [12,13] reported a major QTL on chromosome 2H, a minor QTL on chromosome 7H, and five isolate-specific QTL on chromosomes 2H and 3H. Genome-wide association studies (GWAS) have complemented bi-parental mapping by linking specific genetic markers to barley leaf stripe resistance, further broadening our understanding of resistance loci [14,15]. Collectively, these studies emphasize the complexity of resistance regulation and provide a robust framework for targeted breeding initiatives aimed at developing disease-resistant barley varieties.

In hulless barley, bulk segregant analysis combined with RNA-seq (BSR-seq) was applied to identify resistance-associated genes in a Kunlun 14 × Z1141 population [16]. This approach revealed two genes, *HvnRGA* on chromosomes 5H and *HvnWAK* on chromosome 6H, which were associated with the defense against leaf stripe disease. That study represents an early effort in elucidating the genetic mechanisms underlying resistance to leaf stripe disease in hulless barley.

To expand the resistance resource pool, we evaluated diverse hulless barley germplasm, including landraces, wild accessions indigenous to Tibet, and international varieties [17,18]. Among these, the Tibetan landrace Teliteqingke demonstrated strong resistance to two isolates of *P. graminea* [18]. To explore the genetic basis of this resistance, we crossed Teliteqingke with the susceptible landrace Dulihuang, creating a segregating population for genetic analysis. This study examined the inheritance patterns of resistance and utilized a BSA-SNP array and RNA-seq to identify candidate genes involved in disease resistance. Additionally, quantitative real-time PCR (RT-qPCR) was employed to confirm the differential expression of these candidate genes. This research provides a foundational understanding of the genetic basis of resistance in Teliteqingke and establishes valuable tools for the genetic mapping of leaf stripe resistance genes. Furthermore, it contributes to the integration of these resistance traits into breeding programs, facilitating the development of resilient hulless barley varieties. These efforts support sustainable agriculture and food security on the Qinghai-Tibet Plateau by enhancing the crop’s ability to withstand leaf stripe disease.

## 2. Results

### 2.1. Genetic Analysis of Leaf Stripe Resistance in Teliteqingke

**Figure 1:**
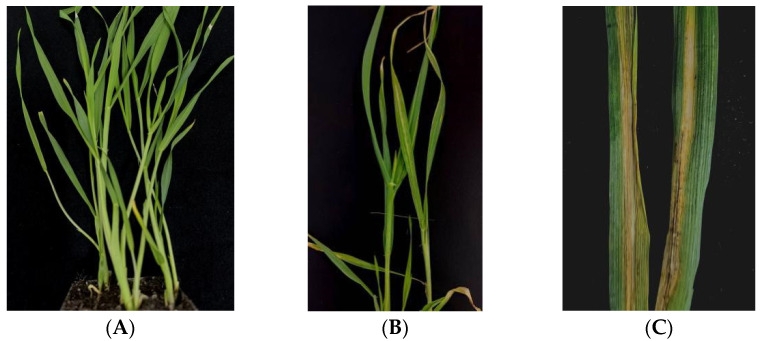
Phenotypic performances of hulless barley landraces Teliteqingke (**A**), Dulihuang (**B**), and typical symptoms of leaf stripe (**C**) caused by *P. graminea* strain FS-18.

We analyzed the inheritance pattern of leaf stripe resistance in Teliteqingke by examining two sets of F_3_ progenies derived from a cross with the susceptible landrace Dulihuang. Both sets were inoculated with the *P. graminea* strain FS-18. Teliteqingke consistently displayed resistance, while Dulihuang was susceptible (Figure 1). In the first test of the segregating population, we observed 141 resistant and 15 susceptible families, which fits a 15:1 segregation ratio (χ^2^ = 1.60, *p* = 0.15). The second test confirmed this segregation pattern, also yielding a 15:1 ratio (χ^2^ = 0.05, *p* = 0.74). These results indicate that the resistance in Teliteqingke is likely governed by two dominant genes, consistent with a digenic inheritance pattern (Table 1).

### 2.2. Identification of Genetic Loci Associated with Leaf Stripe Resistance in Teliteqingke Through BSA-SNP Array

To identify genetic loci conferring resistance, we performed a 40K Genotyping by Target Sequencing (GBTS) SNP array on two contrasting DNA bulks (BulkR and BulkS) and their parental lines. This analysis generated 11.42 Gb of high-quality data, representing over 86% of the raw sequencing data. The sequencing quality was robust, with Q20 (bases with ≤1% error probability) and Q30 (bases with ≤1% error probability) metrics exceeding industry standards (Table 2). A nucleotide composition analysis confirmed a balanced distribution of adenine/thymine (A/T) and guanine/cytosine (G/C) bases, ensuring the accuracy and reliability of downstream analyses. The clean base conversion rates were over 90% for both resistant (BulkR) and susceptible (BulkS) samples and nearly 88% for the parental lines, further validating the dataset’s reliability. These high-quality sequencing results establish a strong basis for identifying genetic loci linked to leaf stripe resistance in Teliteqingke.

**Figure 2:**
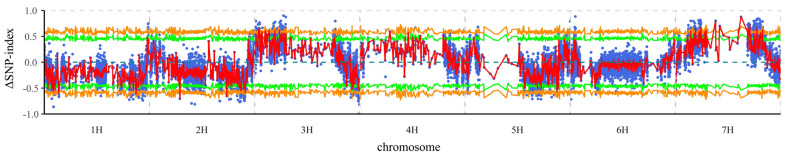
Chromosome distribution in terms of ΔSNP-index correlation value generated by BSA-SNP array.

The alignment rate of clean reads to the reference genome was high for both Teliteqingke (95.55%) and Dulihuang (95.84%), ensuring reliable data for variant analysis. We identified 517,089 SNPs, including 85,821 synonymous mutations (which do not alter amino acid sequences) and 431,268 non-synonymous SNPs (which result in amino acid changes). Additionally, we detected 39,263 insertion or deletion (InDel) sites, where nucleotides are either inserted or deleted. After applying stringent filtering criteria to focus on high-confidence variants, we reduced the dataset to 8745 SNPs and 856 InDels for further investigation. At a 95% confidence level, our refined analysis identified 1376 SNPs and 162 InDels as potential candidates for leaf stripe resistance. Using the SNP-index algorithm, which identifies associations between genetic variants and phenotypic traits, we identified major genetic factors on chromosomes 3H and 7H in Teliteqingke (Figure 2).

### 2.3. Identification of Genetic Loci Associated with Leaf Stripe Resistance in Teliteqingke Through RNA-Seq

To further investigate the genetic factors conferring resistance to leaf stripe disease in Teliteqingke, we conducted an RNA-seq analysis on two contrasting bulks (BulkR and BulkS) and their parental lines. This analysis generated 55 Gb of high-quality data, with Q20 and Q30 scores confirming sequencing accuracy. The GC content was balanced, ranging from 53.22% to 56.32% (Table 3). The sequences mapped to the barley reference genome, MorexV3, with an average rate of 95.54%, indicating reliable data for downstream analysis.

**Figure 3:**
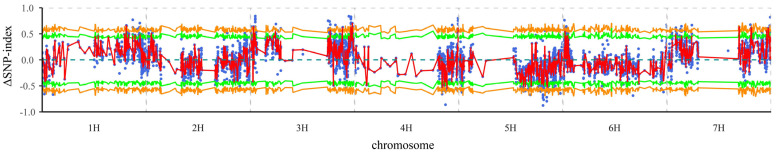
Chromosome distribution in terms of SNP-index correlation value generated by RNA-seq analysis.

From the initial RNA-seq dataset, we detected 192,398 genetic variants, including 164,232 SNPs and 28,166 InDels. Among the SNPs, 134,073 were non-synonymous mutations, which could potentially affect protein function. After applying stringent filtering criteria to improve reliability, we reduced the dataset to 3870 high-quality SNPs and 294 high-quality InDels. Further refinement at a 95% confidence level yielded 298 SNPs and 20 InDels for detailed analysis. Using the SNP-index algorithm, an association analysis identified the primary genetic factors for resistance to leaf stripe disease in Teliteqingke on chromosome 3H (Figure 3).

### 2.4. Co-Localization of Leaf Stripe Resistance-Associated Regions in Teliteqingke

**Figure 4:**
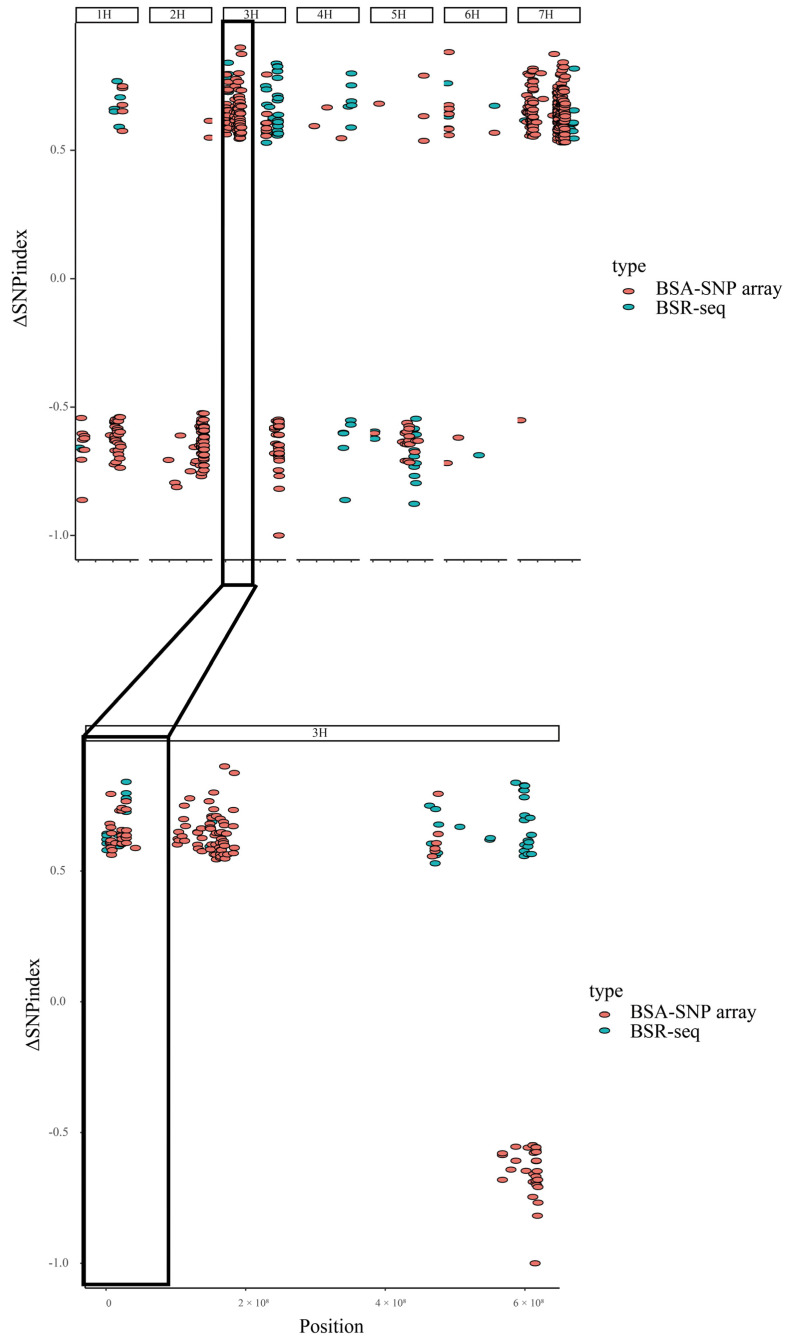
Location of the associated regions of leaf stripe resistance genes.

To elucidate the genetic basis of leaf stripe resistance in Teliteqingke, we conducted an integrated analysis combining BSA-SNP array and RNA-seq data (Figure 4). This approach identified two key candidate regions on chromosome 3H associated with resistance. One region spans a 1.03 Mb interval (28,196,935–29,224,714 bp) and contains 58 genes, while the other spans a 0.19 Mb interval (47,254,165–47,448,374 bp) and included 5 genes.

Within these regions, we identified seven genes that are likely involved in disease resistance. Each encodes proteins with known or putative roles in plant defense mechanisms (Table 4). These include a RING/U-box superfamily protein (*HORVU.MOREX.r3.3HG0232110.1*), which is implicated in ubiquitin-mediated protein degradation, an NBS-LRR disease resistance protein (*HORVU.MOREX.r3.3HG0232120.1*) crucial for pathogen recognition, a cysteine-rich receptor kinase (*HORVU.MOREX.r3.3HG0232550.1*) essential for pathogen perception, an ankyrin repeat protein family-like protein (*HORVU.MOREX.r3.3HG0232140.1*) involved in signal transduction, and an RNA recognition motif (RRM) protein (*HORVU.MOREX.r3.3HG0232180.1*) contributing to pathogen defense responses. Additionally, we found a protease HtpX (*HORVU.MOREX.r3.3HG0232220.1*) and a bZIP transcription factor (*HORVU.MOREX.r3.3HG0232410.1*) associated with stress responses. These results provide a detailed understanding of the genetic architecture underlying leaf stripe resistance in Teliteqingke.

### 2.5. Expression of the Candidate Genes in Response to P. graminea

**Figure 5:**
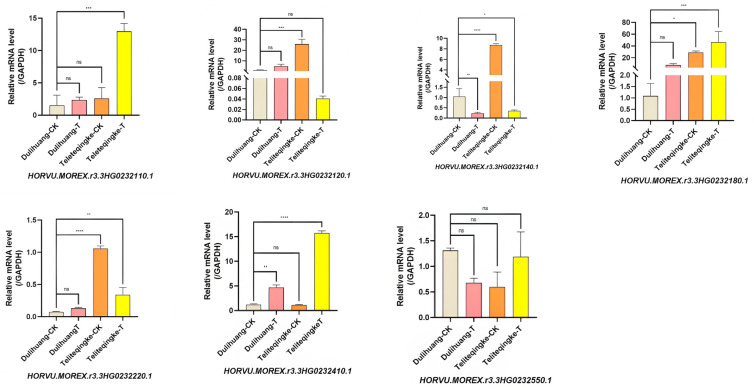
Relative expression of candidate genes in the landraces Teliteqingke and Dulihuang. Teliteqingke-T and Teliteqingke-CK represent the treatment and control groups for Teliteqingke, while Dulihuang-T and Dulihuang-CK represent the treatment and control groups for Dulihuang, respectively. *: *p* < 0.05; **: *p* < 0.01, ***: *p* < 0.001; ****: *p* < 0.0001; ns means no significant difference.

The RT-qPCR analysis of seven disease resistance-associated genes in the resistant landrace Teliteqingke and the susceptible landrace Dulihuang revealed significant differences in expression following inoculation with *P. graminea* (Figure 5). Three genes, *HORVU.MOREX.r3.3HG0232110.1*, *HORVU.MOREX.r3.3HG0232180.1*, and *HORVU.MOREX.r3.3HG0232410.1*, were upregulated in both landraces, but their induction was significantly stronger in Teliteqingke. Specifically, *HORVU.MOREX.r3.3HG0232180.1* and *HORVU.MOREX.r3.3HG0232410.1* exhibited expression levels more than 30-fold and 15-fold higher, respectively, in Teliteqingke compared to Dulihuang, indicating their important roles in active resistance mechanisms.

In contrast, two genes, *HORVU.MOREX.r3.3HG0232120.1* and *HORVU.MOREX.r3.3HG0232140.1*, were induced in the susceptible Dulihuang but suppressed in the resistant Teliteqingke following pathogen infection, indicating differential responses between the two landraces. Additionally, in the absence of infection, *HORVU.MOREX.r3.3HG0232120.1*, *HORVU.MOREX.r3.3HG0232180.1*, and *HORVU.MOREX.r3.3HG0232410.1* were expressed at higher baseline levels in Teliteqingke. This suggests that these genes may contribute to the constitutive defense mechanisms that provide Teliteqingke with its inherent resistance to *P. graminea*.

## 3. Discussion

The resistance of barley to leaf stripe disease arises from complex defense mechanisms shaped by co-evolutionary interactions with the pathogen *P. graminea*. This dynamic interplay determines the plant’s susceptibility or resistance to the disease [19,20]. While previous studies have primarily focused on cultivated barley [9,21], the resistance mechanisms in hulless barley, a crop adapted to the Qinghai-Tibet Plateau, remain underexplored. This study on the hulless barley landrace Teliteqingke provides a new understanding of the genetic basis of leaf stripe resistance in this distinctive crop.

Our genetic analysis revealed that Teliteqingke’s resistance to leaf stripe disease is controlled by two pairs of dominant genes, suggesting a digenic inheritance pattern. The genetic complexity observed demonstrates the need for advanced genomic tools to unravel resistance mechanisms in hulless barley. Using BSA-SNP array technology, we successfully identified chromosomal regions associated with resistance traits on chromosomes 3H and 7H in Teliteqingke, consistent with the inheritance pattern inferred by our genetic analysis. The method of BSA has proven to be effective in identifying candidate genes associated with traits of interest. This method has been widely applied in crops such as wheat (*Triticum aestivum* L.) to identify resistance loci to diseases like powdery mildew and stripe rust [22,23,24,25,26]. It was also used in exploring candidate genes in various crops such as rapeseeds (*Brassica napus* L.), potato (*Solanum tuberosum* L.), hemp (*Cannabis sativa* L.), soybean (*Glycine max* L.), and barley [27,28,29,30,31].

To complement the BSA-SNP array results, we performed RNA-seq analysis to identify the genetic factors linked to leaf stripe resistance in Teliteqingke. RNA-seq analysis enables the identification of differentially expressed genes across various samples [32,33,34]. Data from bulked samples with contrasting resistance phenotypes, along with their parental lines, confirmed the association of specific regions on chromosome 3H with disease resistance. This consistency between BSA-SNP and RNA-seq analyses strengthens the reliability of our results. Integrating data from both methods allowed for the identification of candidate regions on chromosome 3H containing genes potentially responsible for Teliteqingke’s resistance to leaf stripe disease.

Further validation through RT-qPCR confirmed seven candidate genes expressed in both Teliteqingke and the susceptible landrace Dulihuang following inoculation with *P. graminea*. Among these, *HORVU.MOREX.r3.3HG0232110.1* encodes a RING/U-box protein involved in ubiquitin-mediated degradation. The interaction between the RING/U-box E3 protein and the BROAD LEAF1 protein could affect plant leaf sizes [35]. Overexpression of *AtUSR1*, which encodes a plant Ring/U-box protein, accelerates plant senescence in *Arabidopsis* [36]. Another candidate gene, *HORVU.MOREX.r3.3HG0232120.1*, encodes a nucleotide binding site-leucine rich repeats (NBS-LRR) protein, which is a major class of resistance genes and is crucial for pathogen recognition [37]. It was reported that an NBS-LRR gene, *HvtRGA*, was involved in the regulation of resistance to leaf stripe in hulless barley [6]. *HORVU.MOREX.r3.3HG0232550.1* encodes a cysteine-rich receptor kinase, which acts as an upstream signal molecule and is essential in perceiving stress signals and inducing immune responses [38]. *HORVU.MOREX.r3.3HG0232140.1* encodes an ankyrin repeat protein, a common conserved protein domain that serves as a scaffold for protein interactions in various cellular processes [39]. *HORVU.MOREX.r3.3HG0232180.1* encodes an RNA recognition motif (RRM) protein, which is involved in biological processes such as translation, splicing, seed development, and stress signaling in barley [40]. *HORVU.MOREX.r3.3HG0232410.1* encodes a bZIP transcription factor, a family known for its involvement in various plant processes, including flowering induction, stress responses, and defense against pathogens [41]. *HORVU.MOREX.r3.3HG0232220.1* encodes a protease HtpX, and its function remain to be studied.Of these, significant upregulation was observed in Teliteqingke, particularly for *HORVU.MOREX.r3.3HG0232110.1* and *HORVU.MOREX.r3.3HG0232120.1*. The later was expressed at higher baseline levels in Teliteqingke but was not induced in response to pathogen infection.

Additionally, we identified a genomic region on chromosome 7H associated with the leaf stripe resistance in Teliteqingke. This region (28–29 Mb) is distinct from the previously identified *Rdg2a* locus (46.50–46.54 Mb), which encodes a CC-NB-LRR-type protein (*HORVU.MOREX.r3.7HG0636710.1*) [9,42]. These results suggest Teliteqingke employs a combination of active resistance mechanisms and constitutive defenses. Further genetic mapping is needed to confirm the linkage of these genes with leaf stripe resistance. Functional validation using genetic transformation, genome editing, and mutagenesis assays is also required to confirm the roles of these candidate genes.

## 4. Materials and Methods

### 4.1. Plant and Fungal Materials

The leaf stripe-resistant hulless barley landrace Teliteqingke was obtained from the National Crop Germplasm Resource Bank in Xining, Qinghai Province, while the susceptible landrace Dulihuang originated from Gannan Prefecture, Gansu Province. Teliteqingke was crossed with Dulihuang to generate an F_3_ population consisting of 155 lines. Dulihuang served as the susceptible control in the assessment of leaf stripe resistance.

The *P. graminea* strain FS-18 (GenBank ID: PQ452354) was collected from a hulless barley field at the Xining Academy of Agricultural Sciences in Qinghai Province in 2020. It was characterized by morphological observation, phylogenetic analysis with internal transcribed spacer (ITS) sequences, and Koch’s postulates, which confirmed the causal relationship between this strain and the leaf stripe disease [18]. This strain was used to evaluate the segregating population.

### 4.2. Assessment of Disease Resistance

The greenhouse assessment of *P. graminea* resistance was conducted using a method previously described [20]. Briefly, 5 mm diameter plugs of the activated pathogen strain FS-18 were extracted using a cork borer from a pure culture and inoculated into 500 mL conical flasks containing a potato dextrose broth (PDB) medium, with five plugs per flask. The culture was incubated at 25 °C with shaking at 150 rpm for 7–10 min until the mycelium fully colonized the medium.

Teliteqingke was crossed with Dulihuang to generate an F3 population consisting of 155 F3 lines. One set of both parents was inoculated with FS-18 strain and performed with two sets of F3 population for inoculation to ensure experimental reliability. Then, 20 mL of the inoculum was added into Petri dishes. After 48 h of infection, the seeds were transplanted into 5 × 5 cm pots, with 15–20 plants per pot. The plants were grown in an incubator under a 12 h light (20 °C) and 12 h dark (12 °C) cycle. Disease incidence was recorded at 25 days post-seeding using a scale based on infection rates [29]: 0 (immune, I), no infected plant; 1 (high resistance, HR), 0 < incidence rate ≤ 5%; 2 (moderate resistance, MR), 5% < incidence rate ≤ 10%; 3 (moderate susceptibility, MS), 10% < incidence rate ≤ 15%; and 4 (high susceptibility, HS), >15% of infected plants. The disease rate was calculated as follows: disease rate (%) = (number of infected plants/total number of investigated plants) × 100%.

### 4.3. Analysis of BSA-SNP Array

To identify the disease resistance genes in hulless barley landrace Teliteqingke, we performed a BSA analysis. DNA was extracted from the leaves of the two hulless barley parents and their F_3_ lines using the cetyltrimethylammonium bromide (CTAB) method. DNA from 10 resistant and 10 susceptible F_3_ lines was pooled in equal proportions to create the BulkR (resistant) and BulkS (susceptible) pools. Genotyping by Target Sequencing (GBTS) [34] was conducted by MolBreeding Biotech Ltd. (https://en.molbreeding.com). The MorexV3 version of the barley genome, available in the Ensembl Plants database (http://plants.ensembl.org/Hordeum_vulgare, accessed on 15 July 2021), was used as the reference genome.

Sample libraries were sequenced using the Illunima HiSeq 3000 sequencing platform. The resulting reads were filtered to obtain high-quality, clean reads suitable for downstream analysis. Mutations were identified using the UnifiedGenotyper module of the Genome Analysis Toolkit (GATK, version 2.2.1.) [43], and the VariantFiltration module was used to remove unreliable SNP loci and sequence InDels between the test samples and the reference genome.

To identify candidate regions linked to disease resistance traits in Teliteqingke, we calculated the SNP-index for each locus. The SNP-index measures the frequency of SNPs within specific genomic regions. Statistical analysis of changes in the SNP-index [Δ(SNP-index)] was performed to identify genomic regions associated with disease resistance. This methodology enabled comprehensive and accurate mapping of the disease resistance gene in hulless barley [44].

### 4.4. RNA-Seq Analysis

To identify genes related to disease resistance in hulless barley Teliteqingke, we conducted RNA-seq analysis on both resistant and susceptible RNA pools, along with their parental lines. RNA was extracted from the leaves of hulless barley using the Trizol method once full disease symptoms were apparent. The quality and concentration of the RNA samples were assessed using an Ultramicro Spectrophotometer (Machine moder:NO-Onec) to ensure high integrity and absence of contamination. Sequencing was performed on the Illumina HiSeq platform by Beijing Novogene Co. Ltd., Beijing, China. (https://cn.novogene.com, accessed on 15 July 2021). The MorexV3 version of the barley genome (http://plants.ensembl.org/Hordeum_vulgare, accessed on 15 July 2021) was used as the reference genome for analysis.

To evaluate the sequencing data quality, we calculated the base sequencing error rate using Phred scores, which indicate the likelihood of incorrect base calls. We also analyzed the base distribution and filtered the raw reads to generate high-quality, clean reads for further analysis. Clean reads from Teliteqingke and Dulihuang were aligned to the MorexV3 reference genome using Hisat2 (version 2.2.1). SNPs and InDels were identified using the Genome Analysis Toolkit (GATK, version 2.2.1.) [43]. After filtering, the SNP-index algorithm was applied to conduct association analysis and identify genetic loci linked to disease resistance.

### 4.5. Screening and Verification of Genes Related to Leaf Stripe Resistance in Teliteqingke

To identify genes associated with leaf stripe resistance in Teliteqingke, we conducted an RT-qPCR to measure gene expression in response to *P. graminea* inoculation. Two-week-old seedlings of Teliteqingke and Dulihuang were inoculated with the *P. graminea* strain FS-18 as treatment groups, while mock-inoculated seedlings served as the control groups. Each group included three biological replicates and three technical replicates. Inoculated plants were incubated in a controlled environment with a 12 h light cycle and a temperature alternating between 20 °C and 12 °C.

RNA was extracted from the leaves using the Trizol method, and cDNA was synthesized with the Prime Script™ RT reagent Kit with gDNA Eraser (TaKaRa, Beijing, China). The reverse transcription reaction was conducted at 37 °C for 15 min, followed by incubation at 85 °C for 5 s, and stored at 4 °C. The synthesized cDNA was preserved at −20 °C for long-term storage.

Candidate gene sequences were retrieved from the barley reference genome based on their identification (ID) codes. RT-qPCR primers, listed in Table 5, were designed using Primer premier5 software (https://www.premierbiosoft.com/primerdesign, accessed on 7 April 2024) and synthesized by Qinghai Kemeiyi Biotechnology (Xining, China). RT-qPCR was performed using TB Green^®^ premix Ex Taq™ II fluorescent Dye and ROX Reference Dye (TaKaRa, Beijing, China), with *18SrRNA* serving as the internal reference gene. The reaction mixture (20 µL) consisted of 10.0 µL of the TB Green^®^ premix Ex Taq™ II (Tli RNaseH Plus) (2×), 0.8 µL of PCR forward and reverse primers each (10 µmol/L), 0.4 µL of ROX Reference Dye (50×), 2.0 µL of cDNA template, and 6.0 µL of RNase free dH_2_O. The amplification conditions included an initial denaturation at 95 °C for 10 min, followed by 40 cycles of 95 °C for 15 s, 60 °C for 30 s, and 72 °C for 30 s. Relative gene expression levels were calculated using the 2^−ΔΔCt^ method [45].

### 4.6. Statistical Analysis

A Chi-squared test was used to determine the goodness of fit for the segregation ratio. Data on relative gene expression levels were subjected to analysis of variance (ANOVA), and the significant difference was determined using Fisher’s least significant difference (LSD) method at *p* < 0.05 or *p* < 0.01). All statistical analyses were performed using GraphPad Prisim8.4.3 software (https://www.graphpad.com, accessed on 5 May 2024).

## 5. Conclusions

Our study advances the understanding of the genetic basis of leaf stripe resistance in the hulless barley cultivar Teliteqingke. By integrating the BSA-SNP array and RNA-seq technologies, we identified a candidate region on chromosome 3H linked to resistance, and detected seven candidate genes likely involved in this resistance mechanism. These results contribute to a deeper understanding of the genetic factors conferring resistance to leaf stripe disease and offering valuable targets for future breeding efforts in hulless barley. Further research is needed to validate the function of these candidate genes using genetic and molecular biological approaches, such as genetic transformation, genomic editing, and other molecular approaches. To facilitate the use of resistance in Teliteqingke for cultivar development, the identification of linked or functional molecular markers will be essential for molecular marker-assisted selection, enabling the efficient transfer of resistance genes to other hulless barley cultivars. Ultimately, this work provides breeders with important genetic resources to improve disease resistance in hulless barley.

## Figures and Tables

**Table 1:** Genetic analysis of leaf stripe resistance gene in hulless barley landrace Teliteqingke.

Parents and Cross	Generations	Total Number of Plants/Lines	No. of Resistant Plants/Lines	No. of Susceptible Plants/Lines	Expected Ratio		χ^2^	*p*-Value
Teliteqingke	P1		15					
Dulihuang	P2			15				
P1 × P2	F_3_	155	141	14	15	1	1.60	0.15
P1 × P2	F_3_	155	137	18	15	1	0.05	0.74

**Table 2:** Quality statistics of sequencing data from the 40K GBTS SNP array.

Sample	No. of Raw Reads	No. of Clean Reads	Raw Bases (bp)	Clean Bases (bp)	Effective Rate (%)	Q20 (%)	Q30 (%)
Teliteqingke	23,712,526	23,675,688	3,556,878,900	3,134,944,054	88.14	97.65	93.00
Dulihuang	20,508,626	20,484,396	3,076,293,900	2,672,254,594	86.87	97.67	93.10
BulkR	23,028,646	22,986,476	3,454,296,900	3,122,676,470	90.40	97.41	92.32
BulkS	18,231,610	18,206,664	2,734,741,500	2,502,101,400	91.49	97.42	92.32

**Table 3:** Quality statistics of RNA-seq analysis.

Sample	Raw Reads	Clean Reads	Clean Bases (Gb)	Error Rate (%)	Q20 (%)	Q30 (%)	GC Content (%)	Alignment Rate (%)
Dulihuang	91,293,194	90,303,276	13.55	0.03	97.81	93.62	54.32	95.84
Teliteqingke	90,722,940	90,073,344	13.51	0.03	97.84	93.72	56.32	95.55
BulkS	96,945,580	96,231,078	14.43	0.03	97.62	93.13	56.17	95.31
BulkR	92,230,542	91,393,264	13.71	0.03	97.72	93.39	53.22	95.45

**Table 4:** Functions of seven candidate genes on chromosome 3H associated with plant defense mechanisms.

Gene ID	Start	End	Description
*HORVU.MOREX.r3.3HG0232110.1*	2,841,496	284,145,536	RING/U-box superfamily protein
*HORVU.MOREX.r3.3HG0232120.1*	28,429,178	28,431,928	NBS-LRR disease resistance protein
*HORVU.MOREX.r3.3HG0232140.1*	28,461,280	28,455,885	Ankyrin repeat protein family-like protein
*HORVU.MOREX.r3.3HG0232180.1*	28,520,789	28,515,702	RNA recognition motif (RRM) containing protein
*HORVU.MOREX.r3.3HG0232220.1*	28,691,124	28,689,091	Protease HtpX
*HORVU.MOREX.r3.3HG0232410.1*	28,902,070	28,897,759	bZIP transcription factor; putative (DUF1664)
*HORVU.MOREX.r3.3HG0232550.1*	29,213,059	29,213,793	Cysteine-rich receptor kinase

**Table 5:** Candidate gene primers (RT-qPCR).

Gene ID	Forward Primer (5′-3′)	Reverse Primer (3′-5′)
*HORVU.MOREX.r3.3HG0232110.1*	GATGATAAGCCCGCCATAGA	CCGATGTCCACATGGTAAGA
*HORVU.MOREX.r3.3HG0232120.1*	CCAAGCACTCAAGCCAATTTC	CTTCCCATGACCCTGGAATATC
*HORVU.MOREX.r3.3HG0232140.1*	GGAGGTTCACTCACATGCTTAT	CACAACACCACAAGAGGACTAA
*HORVU.MOREX.r3.3HG0232180.1*	GCGGATGAAACTGGTACAGATA	CATTAATGTCGGACACGGTAGA
*HORVU.MOREX.r3.3HG0232220.1*	GTCACCTCAAGTGCGATCAT	AAGGAACCCAGCAACCATAC
*HORVU.MOREX.r3.3HG0232410.1*	CGTGTCTCCTGTAGCTCAATC	CTCAGTCCTGATGCTGATATGG
*HORVU.MOREX.r3.3HG0232550.1*	ATCTGACCACTACACCAAACC	CTCACCTTTCGTAGCCTTGAA

## Data Availability

The data presented in this study are available in this article.
